# Synergistic Effects and Mechanism of Modified Silica Sol Flame Retardant Systems on Silk Fabric

**DOI:** 10.3390/ma11101842

**Published:** 2018-09-27

**Authors:** Chun Liu, Tieling Xing, Bingju Wei, Guoqiang Chen

**Affiliations:** National Engineering Laboratory for Modern Silk, College of Textile and Clothing Engineering, Soochow University, Suzhou 215123, China; 15106135639@163.com (C.L.); m18896936103@163.com (B.W.); chenguojiang@suda.edu.cn (G.C.)

**Keywords:** silk, flame retardancy, sol-gel processes, synergisms

## Abstract

The nano-silica sol was prepared by sol-gel method, and the boric acid, urea, cyanoguanidine, melamine cyanurate (MCA), 1-hydroxyethane 1,1-diphosphonic acid (HEDP), and 6H-dibenz (C,E) (1,2) oxaphosphorin-6-oxide (DOPO) were added to the silica sol to modify the flame retardant through physical doping and chemical bonding. According to the formula proposed by Lewin, the calculation of flammability parameters were obtained by the limiting oxygen index meter, the micro calorimeter, the vertical burner, and the thermogravimetric analyzer proved that there was a synergistic or additive effect between the B/N/P flame retardant and the silica sol. Fourier transform infrared (FT-IR) spectrum, scanning electron microscopy, and pyrolysis gas chromatography-mass spectrometry were used to characterize the morphology, structure, and pyrolysis products of treated silk fabric and residues after combustion. The results show that the flame retardancy of silica-boron sol is mainly caused by endothermic reaction and melt covering reaction. Silicon-nitrogen sol acts as a flame retardant through endothermic reaction, release of gases, and melting coverage. Silicon-phosphorus sol achieves flame retardancy by forming an acid to promote formation of a carbon layer and melting coverage. Silica sol and other flame retardants show excellent flame retardanty after compounding, and have certain complementarity, which can balance the dosage, performance, and cost of flame retardants, and is more suitable for industrial development.

## 1. Introduction

Silk is one of the important natural fibers in the textile field. It has the advantages of biocompatiblity, biodegradablity, gentle luster, good hygroscopicity, excellent mechanical properties, and comfortable handle [1]. Silk products are mostly used in pajamas, ties, silk quilts, decorations, etc., and most of them are in direct contact with the human body [2]. According to the survey, the proportion of textiles caused by fire accidents is more than 20%, and more than half of the deaths are related to these fires [3]. The limiting oxygen index (LOI) of silk fibers is about 23.1%, which is a flammable fiber [4]. Therefore, it is very practical to carry out flame retardant finishing to improve its fire safety performance and meet the needs of the international market.

As a natural fiber, silk cannot be obtained by blending the spinning solution with the flame retardant in the spinning stage like synthetic fibers. The flame retardancy of silk is generally achieved through finishing technology. Conventional finishing processes mainly include impregnation drying and pad-dry-cure method. At present, some emerging technologies such as graft copolymerization modification [5], layer-by-layer assembly [6,7], and sol-gel technology [8] have been increasingly used in the flame retardant finish of silk fabrics.

There are many common flame retardants, such as boron flame retardants, halogen flame retardants (gradual elimination), phosphorus flame retardants, nitrogen flame retardants, silicon flame retardants, phosphazene flame retardants, etc. [9]. Among the silicon-based flame retardants, silica sol is commonly used for textiles finishing. With the continuous development of science and technology, the application scope of silica sol has gradually expanded and the demand for use has gradually increased. The silica sol containing single flame retardant element cannot meet the development demand of industry. The single flame retardant has the problems of high price and poor flame retardant effect in use. In recent years, the flame retardant synergy system has been widely studied, and there are two concepts of synergy [10,11]: (1) When two or more flame retardants are used together, the flame retardant effect is better than that of single; and (2) when flame retardant is used alone, adding other materials (non-flame retardants, such as acrylamide [12]) to the flame retardant system, the flame retardant effect is also improved accordingly.

Jenny Alongi et al. [13,14] studied the silica sol prepared from five different structural precursors, and the effect of silica sol doped with different flame retardants, such as ZnO, ZnB and ammonium polyphosphate (APP), on the flame retardancy of the finished cotton fabric were also investigated. QH Zhang et al. [15] improved the flame retardant properties of wool fabrics by doping boric acid into a silica sol prepared using tetraethyl orthosilicate (TEOS) as a precursor. J Vasiljevic et al. [16] prepared a fluorine-containing, phosphorus-containing and nitrogen-containing composite sol by a sol-gel technique. This flame retardant system consisting of two (flame retardant, synergist) or more flame retardant components is called synergistic flame retardant, and its flame retardant effect is superior to that of single component flame retardant [17]. 

Like other textiles and combustible materials, the combustion of silk fabrics requires three elements: Combustible materials, heat, and oxygen. The combustion of silk can generally be divided into three processes: Thermal oxygen degradation of protein macromolecular chains (Process I), aerobic combustion of small molecule degradation products (Process II), and generated thermal feedback (Process III) [18,19]. Therefore, as long as any process in the combustion cycle of the silk fibers is suppressed or destroyed, the purpose of flame retardancy can be achieved. For example, in the process of heating the silk fibers, the heat released from the combustion is preferentially absorbed by the endothermic degradation of the flame retardant. Reducing the energy of the system in the silk burning Process I can achieve the purpose of delayed combustion. Some flame retardants are thermally decomposed and generate free radicals, which can interrupt the strong free radical chain reaction and inhibit further combustion of small molecules degradation products. Reducing the energy released by the combustion system in Process II can achieve the effect of suppressing combustion. This can also be done by diluting the flammable gas. The flame retardant can generate a large amount of incombustible gas during the thermal decomposition process [20], reducing the heat source in Process III to achieve the purpose of flame retardancy. In addition, some flame retardants melt while they degrade and absorb heat, forming a dense molten coating on the surface of silk fibers [21], thereby inhibiting the II and III processes in the combustion cycle of the silk. Other flame retardants will form acid or alkali during the thermal decomposition process, which will dehydrate the fibers to form a non-volatile carbon layer [22]. The carbon layer can directly block the generation of flammable volatiles in the combustion Process II and inhibit the thermal cracking reaction in Process I to some extent.

In this work, the boric acid, urea, cyanoguanidine, melamine cyanurate (MCA), 1-hydroxyethane 1,1-diphosphonic acid (HEDP) and 6H-dibenz (C,E) (1,2) oxaphosphorin-6-oxide (DOPO) were added to the silica sol to improve the flame retardancy of the silk fabric. At the same time, the synergetic index was calculated from the flammability parameters obtained by the oxygen index meter, the micro calorimeter, the vertical combustion, and the thermogravimetric analyzer, confirming the synergistic and additive effects of different elements (B, N, P) flame retardants with silica sol. By utilizing the synergistic effect of flame retardants to compensate for the shortage of single flame retardant method, the relationship between the amount of flame retardants, performance, and cost can be better balanced, and the increasing requirements of environmental protection and safety can be met. In order to further explore the action mechanism of these flame retardants, FT-IR infrared spectroscopy, scanning electron microscopy, and pyrolysis gas chromatography-mass spectrometry were used to illustrate the morphology, structure, and cleavage products.

## 2. Materials and Methods

### 2.1. Materials

Silk fabrics (11206, habotai, 43 g/m^2^) were supplied by Suzhou Huajia Group of Suzhou, China; the silica precursor (tetraethyl orthosilicate, T, analytical grade) was provided by Sinopharm Chemical Reagent Co., Ltd., Shanghai, China; anhydrous ethanol (analytical grade), and hydrochloric acid (excellent grade) were provided by Jiangsu Qiangsheng Functional Chemical Co., Ltd., Suzhou, China; Boric acid (B, 99.5%), urea (N_1_, 99%), cyanoguanidine (N_2_, 99.5%), HEDP (P_1_, 60% aqueous solution), and DOPO (P_2_, 97%) were purchased from Sinopharm Chemical Reagent Co., Ltd., Shanghai, China; MCA (N_3_, nanometer) was supplied by Zhengzhou Guanda Chemical Products Co., Ltd., Zhengzhou, China. The chemical structure of Si, B, N (N_1_, N_2_, N_3_), and P (P_1_, P_2_) are reported in Table S1.

### 2.2. Methods

52 g (0.25 mol) of TEOS and 15.3 g (0.33 mol) of absolute ethanol (EtOH) was put in a 250 mL three-neck round bottom flask, and the condensing device was open to set the constant temperature heating magnetic stirrer at 70 °C, and 12.71 g of boric acid solution with pH of about 2 was slowly added to the three-necked flask while stirring with a peristaltic pump. The molar ratio of Si/B was 1:0.24, and the mixture was heated to 80 °C for 3 h to obtain a boric acid-modified silica sol, and the concentration of the sol (Si + B) was 3 mol/L. 52 g (0.25 mol) of TEOS, 15.3 g (0.33 mol) of EtOH, and 3.79 g (0.06 mol) of urea (N_1_)/2.63 g (0.03 mol) of cyanoguanidine (N_2_)/14.18 g (0.06 mol) of MCA (N_3_) were added to a 250 mL three-neck round bottom flask, and the condensing device was turned on to set the constant temperature heating magnetic stirrer at 70 °C. Deionized water having a pH of about 2 was slowly added to the three-necked flask while stirring with a peristaltic pump, wherein Si:N_1_ = 2:1, Si:N_2_ = 2:1, Si:N_3_ = 1:2, Si:P_2_ = 3:1 (molar ratio of elements). Each sol was stirred for 3 h, and the concentration of the sol sol was 3 mol/L (Si + N_1,2,3_ = 3). 85.8 g (0.25 mol) of HEDP (P_1_) was added to silica sol, and shaken in an ultrasonic apparatus to uniformly mix, wherein the molar ratio of Si/P element was 1:2 and the concentration of the sol (Si + P_1_) was 3 mol/L. 52 g (0.25 mol) of TEOS, 23 g (0.5 mol) of EtOH, and 18 g (0.08 mol) of DOPO (P_2_) were added to a 250 mL three-neck round bottom flask (the molar ratio of Si/P_2_ was 3:1). The condensing unit was open to set the stirrer at 70 °C. Deionized water (pH = 2 to 3) was slowly added dropwise to the flask using a peristaltic pump and stirred for 3 h, and the sol was determined to 3 mol/L (Si + P_2_). The fabrics treated with Si and B, Si, and N_1_ or N_2_ or N_3_, Si, and P_1_ or P_2_ sol were represented as Si-B, Si-N_1_ and S-N_2_, Si-N_3_, Si-P_1_, and Si-P_2_, respectively.

In order to calculate the “synergistic effect”, the fabrics were also treated with only Si, B, N_1_, N_2_, N_3_, P_1_, P_2_ (Silk-Si, Silk-B, Silk-N_1_, Silk-N_2_, Silk-N_3_, Silk-P_1_, Silk-P_2_ samples, respectively), and the following procedure was described below. 52 g (0.25 mol) of TEOS and 23 g (0.5 mol) of EtOH were sequentially added to a 250 mL three-neck round bottom flask, and 5.94 g (0.33 mol) of deionized water was added while stirring. Wherein, the molar ratio of each substance was n [TEOS]:n [EtOH]:n [H_2_O] = 1:2:1.33. HCl was added to adjust pH to 2–3, and the solution was continuously stirred at 70 °C for 3 h to obtain a uniform colorless transparent silica sol. The silica sol with 3 mol/L of Si content was finally made. Boric acid, urea, cyanoguanidine, hydroxyethylidenediphosphonic acid, and DOPO were dissolved in deionized water at a certain temperature and the volume of B, N, and P was 3 mol/L. The nano-sized melamine cyanurate was stirred with anhydrous ethanol at 120 °C for no less than 30 min until it becomes a homogeneous mixture, and the N content was also 3 mol/L.

**Scheme 1:**
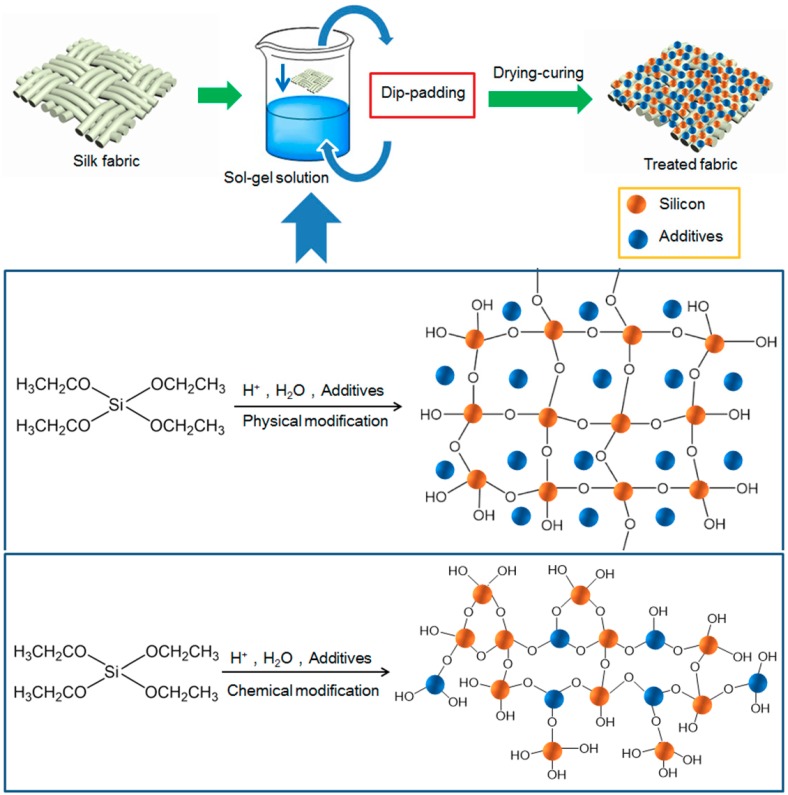
The schematic diagram of the sol-gel treatments on silk fabrics and the modification of sol-gel.

All silk fabrics were prepared with the same process, as shown in Scheme 1. Silk samples were immersed in the prepared solution/sol with 1:30 of liquor ratio, and underwent two dipping and two padding (each dipping 5 min, the padding pressure was 0.4 mPa), then dried at 100 °C for 4 min and cured at 160 °C for 2 min. After washing the samples with de-ionized water, it was placed in an oven at a temperature of 60 °C until it was dried. Then the samples were stored for use.

The total dry solids add-on on silk samples (A, wt.%) was calculated according to the following equation:

(1) $$\;A\left(\%\right)=\frac{W_{f}-W_{i}}{W_{i}}\;\;\;$$

where (*W_i_*) is the weight before treatment, (*W_f_*) is the weight after the impregnation with the sols and subsequent thermal treatment.

Lewin [23] and Horrocks [24] proposed a formula that calculates the synergistic effect (SE) parameter to determine the true level of synergy between the two species.

The calculation formula of SE [23] is as follows:

(2) $$\;\mathrm{SE}=\frac{\begin{array}{c} {\left(Fp\right)}_{fr+s}-{\left(Fp\right)}_{p} \end{array}}{\left[{\left(Fp\right)}_{fr}-{\left(Fp\right)}_{p}\right]+\left[{\left(Fp\right)}_{s}-{\left(Fp\right)}_{p}\right]}\;$$

where (*Fp*) is the parameter obtained from flammability or combustion tests. (*Fp*)*_p_* is thepolymer alone, (*Fp*)*_fr_* is that of the polymer and flame retardant, (*Fp*)*_s_* is that of the polymer treated with the synergist, and (*Fp*)*_fr+s_* is thatof the full formulation comprising flame retardant and synergist. When SE > 1, there is a synergistic effect between the two substances; when 0 < SE < 1, there is only a simple addition or accumulation effect between the two substances, and there is no synergistic effect; when SE < 0, it means that there is a mutual counteraction between the two substances [25].

### 2.3. Sample Characterizations 

Scanning electron microscopy (SEM) was conducted using a TM-3030 (Hitachi, Tokyo, Japan) instrument in order to observe the surface morphology of the flame retardant silk fabrics before and after burning. All the samples were sputter-coated with a conductive gold layer before SEM analysis. At the same time, the energy-dispersive X-ray spectroscopy (EDS) (Hitachi, Tokyo, Japan) was used to nearly quantitatively characterize the element distribution of the micro-area of the silk fabric before and after finishing. The test conditions were as follows: Acceleration voltage 3.0 kV, and magnification 2.0 k.

The LOI determination was performed according to ASTM D-2863 [26] using the FTT0080 Oxygen index apparatus (Fire Testing Technology Ltd., East Grinstead, UK). The vertical burning test was carried out on a YG851B automatic vertical flammability cabinet (Nigbo Textile Instrument Factory, Ningbo, China). The samples were exposed to 40 ± 2 mm vertical flame for 12 s, and the damage length was measured. The test was repeated 3 times for each sample.

The heat release performance was measured by the FTT001 miicroscale combustion calorimeter (MCC, Fire Testing Technology Ltd., East Grinstead, UK) [15]. The flow was a mixture of O_2_/N_2_ 20/80 cm^3^·min^−1^, and the sample weight was 5.0–6.0 mg. The heating rate is 1/s and the temperature range is 75–750 °C. All experiments were performed in triplicate, and heat release rate (HRR) can be repeated to within ±5%.

The thermal stability of the silk fabric was evaluated by thermogravimetric (TG) analysis with the SDT2960 thermal analyzer (Perkin-Elmer, Waltham, MA, USA). The samples of 4–5 mg were obtained in crucible and heated in a nitrogen atmosphere (100 mL/min). The temperature was set from 30 °C to 700 °C at the scan rate of 10 °C/min.

The samples were tested using a PY-2020iD double-click splitter (Frontier Laboratories Ltd., Osaka, Japan) and a QP2010 gas chromatography-mass spectrometer (Shimadzu Ltd., Kyoto, Japan). Based on the thermogravimetric analysis results, the pyrolysis temperature in the experiment was set to 570 °C. The chemical structure of the pyrolysis product was searched and determined using the Shimadzu IST11 mass spectrometer library (Shimadzu Ltd., Kyoto, Japan). The relative content of the pyrolysis product was calculated by normalizing the measured area of the peak on the total ion chromatogram.

The Fourier transform infrared spectra of the samples were measured on a Nicolet5700 FT-IR spectrometer (Thermo Fisher Scientific Inc., Waltham, MA, USA) using KBr pellets and the results were collected from 120 scans.

The smoke suppression of the samples analysis was conducted using an NBS smoke density test chamber (Fire Testing Technology Ltd., East Grinstead, UK) for 600 s with max radiant heat of 50 kW/m^2^ and three sample layers of 80 mm × 80 mm.

## 3. Results and Discussion

Table S2 lists the add-on values of treated silk fabric corresponding to each formulation. As shown in Table S2, the total dry solids add-on on the binary sol-treated silk (Si-B, Si-N_1_ and S-N_2_, Si-N_3_, Si-P_1_ and Si-P_2_) is obviously higher than that of the single flame retardant treated silk (Silk-Si, Silk-B and Silk-N_1_, Silk-N_2_, Silk-N_3_, Silk-P_1_, and Silk-P_2_), with the maximum difference of 23.25%. This is because the sol-gel system has a certain adsorption property on silk, and can form a certain film on the surface of the fabric, so that more modified sol finishing agent adheres to the silk surface.

### 3.1. Morphology 

**Figure 1:**
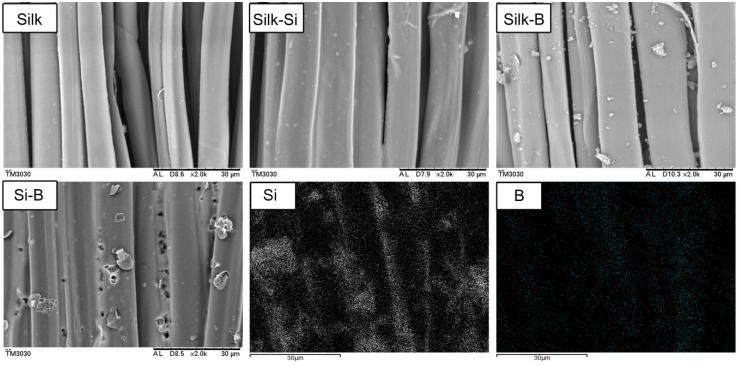
SEM magnifications of Silk, Silk-Si, Silk-B, Si-B samples, and elemental analysis of Si-B sample.

**Figure 2:**
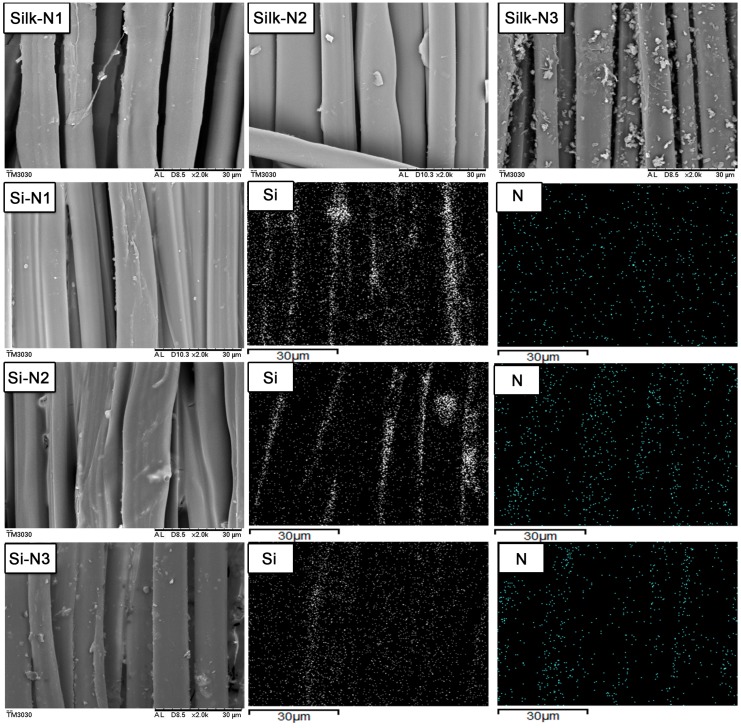
SEM magnifications of Silk-N_1_, Silk-N_2_ and Silk-N_3_, Si-N_1_ and Si-N_2_, Si-N_3_ samples and elemental analysis of Si-N_1_, Si-N_2_ and Si-N_3_ samples.

**Figure 3:**
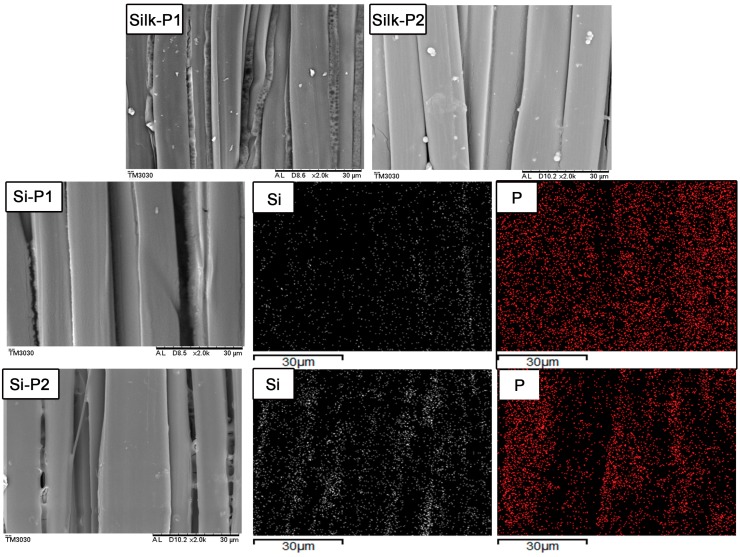
SEM magnifications of Silk-P_1_, Silk-P_2_ and Si-P_1_ and Si-P_2_ samples and elemental analysis of Si-P_1_, Si-P_2_ samples.

SEM observations have been performed in order to observe the morphology of the fibers after the various treatments. As depicted in Figure 1, Figure 2 and Figure 3, the original silk fabric (Silk) has a smooth surface, no obvious cracks and sediments, and there are gaps between the fibers. The silk fabric finished with pure silica sol (Silk-Si) is coated with thin film. This is a network structure in which SiO_2_ sol particles can be dehydrated and condensed at high temperature, and the coating on the fibers surface is very complete. However, the surface of silk fabrics finished with boric acid, urea, cyanoguanidine, MCA, HEDP, or DOPO (Silk-B, Silk-N_1_, Silk-N_2_, Silk-N_3_, Silk-P_1_, and Silk-P_2_, respectively) are not smooth and are attached with fine particles. The surface of the fabric modified by the compounding of the silica sol (Si-B, Si-N_1_ and S-N_2_, Si-N_3_, Si-P_1_, and Si-P_2_, respectively) is smoother than that of the flame retardant alone, and the attached fine particles are greatly reduced. It is also seen that there is a film covering, indicating that the flame retardant has been successfully coated into the silica sol film. Through the EDS results (Figure 1, Figure 2 and Figure 3), the distribution of the elements on the Si-B, Si-N_1_ and Si-N_2_, Si-N_3_, Si-P_1_, and Si-P_2_ fabric are relatively uniform. 

While the EDS investigation is only a semi-quantitative analysis, by employing a high beam voltage (i.e., 20 kV), the high penetration of the incident beam can be utilized to discriminate the depositions obtained with all of the different dopants. All data (the average of 5 results) are listed in Table 1.

### 3.2. Flame Retardancy 

The limiting oxygen index (LOI) and the vertical burner test were used to evaluate the flame retardancy of the treated silk fabrics. As shown in Table 2, although different flame retardants have different effects on the flame retardancy of silk fabric, the LOI of the treated silk fabric is obviously improved compared with the original silk (LOI is 24) and the length of the vertical burning is also reduced. These flame retardants have different mechanisms for the combustion performance change of silk. Among them, the intumescent flame retardant, such as silica sol, can form a carbon foam layer on the fabric surface, and has the flame retardant effect of heat insulation, smoke suppression, oxygen barrier, and prevention of droplet formation.

**Figure 4:**
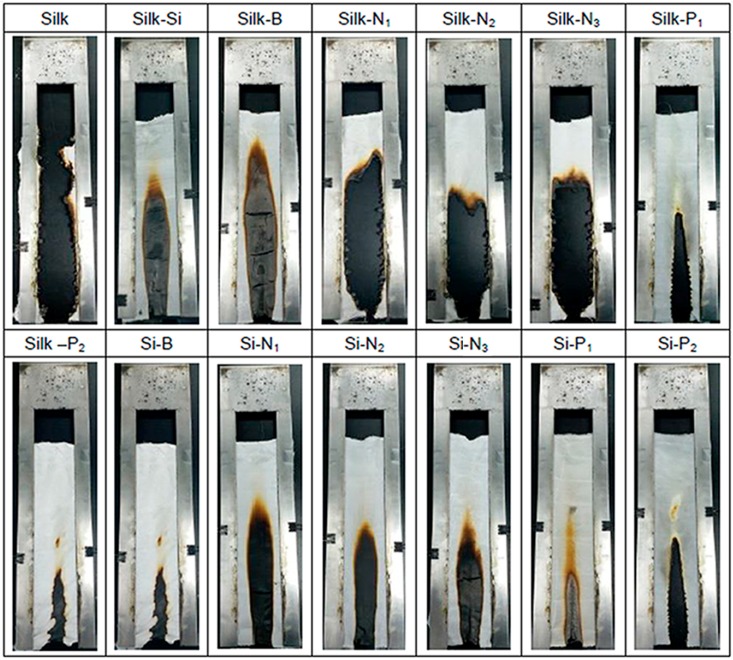
Vertical burning images of original silk and the treated silk fabrics.

It can be concluded from Table 2 that the Si-B sol, the Si-N sol, and the Si-P sol have a relatively significant effect on the improvement of the LOI of silk fabric, wherein the lowest LOI is 28.5 and the highest is 38.6. In addition to Si-P_2_, the other five groups of compounds (Si-P_1_, Si-N_1_ and S-N_2_, Si-N_3_ and Si-P_1_) treated silk fabric have higher LOI than that of silk treated with single flame retardant (Silk-Si, Silk-B and Silk-N_1_, Silk-N_2_, Silk-N_3_, and Silk-P_2_). In the vertical burning test, the damaged length of the original silk is 30 cm (completely damaged). In addition to Si-N_1_ and Si-N_2_, the damaged length of the other four sets of compound flame retardants treated silk fabric are obviously lower than those of the corresponding single element flame retardant. Among them, the damaged lenght of silk finished by Si-B and Si-P_1_ was reduced to less than 10 cm, with a drop of more than 2/3. Figure 4 shows the vertical burning images of original silk and the treated silk fabrics.

According to the synergistic effectiveness (SE) calculation method given by Lewin [23] in the preface, the SE of LOI and the carbon length were obtained. As shown in Table 2, SE (Si-B), SE (Si-N_1_), SE (Si-N_3_) and SE (Si-P_2_) results of LOI are greater than one, which means the LOI SE of Si-B, Si-N_1_, Si-N_3_ and Si-P_2_ are not simply additive effects, but synergistic effects with SE (Si-N_3_) > SE (Si-N_1_) > SE (Si-B) > SE (Si-P_2_) of order. As 0 < SE (Si-P_1_) < SE (Si-N_2_) < 1, Si-N_2_ and Si-P_1_ are simply cumulative effects without synergy. In terms of carbon-damaged length, combining with the SE obtained by LOI, SE (Si-B), SE (Si-N_1_), and SE (Si-N_3_) still have obvious synergistic effects, and Si-P_1_ and Si-P_2_ also show weak synergistic effects.

**Figure 5:**
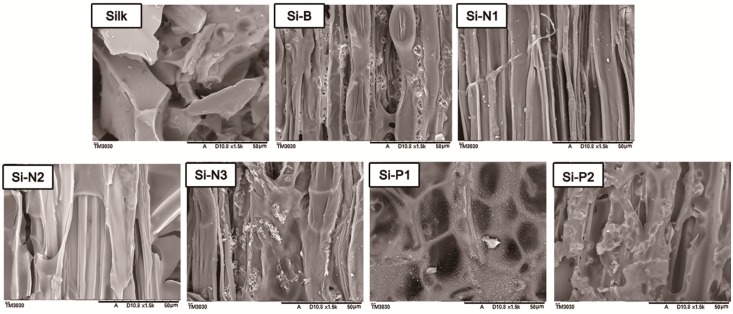
SEM micrographs of the residues left by Silk, Si-B, Si-N_1_ and Si-N_2_, Si-N_3_, Si-P_1_ and Si-P_2_ after the flammability tests.

As shown in Figure 5, the untreated silk (Silk) loses its original fibers shape and arranged loosely after being subjected to the vertical burning treatment. The surface of the finished fabrics after being subjected to the vertical burning treatment (Si-B, Si-N_1_ and Si-N_2_, Si-N_3_, Si-P_1_, and Si-P_2_) shows obvious changes: The residual carbon residue is high and closely arranged, and the uniformity is higher, indicating that the fabric finished with the modified silica sol has a layer of thin film. During the combustion process, it acts as a melting cover, melting while absorbing heat, forming a dense molten coating on the surface of silk fibers [27]. Therefore, the infiltration of oxygen during combustion and the release of heat and flammable gases can be prevented, inhibiting the II and III processes in the combustion cycle of silk fibers.

### 3.3. Thermal Analysis

The micro calorimeter (MCC) can fully characterize the thermal release behavior of materials during combustion [28]. Table 3 presents the MCC results. As can be seen from Table 3, the finished silk fabrics basically exhibit milder heat release behavior. The modified silica sol has a lower heat release capacity (HRC), peak heat release rate (pHRR), and total heat release (THR) than the original silk and silk treated by single element flame retardant. Among them, the Si-P_2_ sample showed the best heat release performance in all samples with 57 J/g·k of HRC, 45.3 W/g of pHRR and 9.8 kJ/g of THR. It seems the heat release performance of the phosphorus-silica sol (Si-P_1_, Si-P_2_) are quite good. Compared with the silicon-boron sol and the silicon-nitrogen sol, the HRC, pHRR, and THR of the phosphorus-based flame retardant are low when separately sorted, showing that there is no good synergy between silicon and phosphorus in the sol system. However, through the MCC test data, it can be stated that the phosphorus-based flame retardant is condensed phase flame retardant. It generates acid (phosphoric acid or polyphosphoric acid) during the thermal decomposition process, which dehydrates and softens the silk fibers to form a non-volatile carbon layer [29]. The carbon layer can directly prevent the generation of flammable volatiles in the combustion process II, and the thermal pyrolysis reaction in Process I is inhibited to some extent.

In the SE data of MCC (excluding T_max_), only SE (Si-B) > 1, that is, there is synergistic effect between Si-B sol. Si-N sol, Si-P sol (the SE_(THR)_ of Si-P_1_ is greater than one) are only additive effect (0 < SE < 1). It means that Si-B not only has synergistic effect on improving the flame retardancy of silk fabric, but can also reduce the heat release rate of fabric combustion. As a result, the risk of fabric burning is reduced. The synergistic effect between Si-B may be due to the fact that both Si and B can cover the silk fabric during the combustion process (the former forms a carbon layer and the latter melts). Accordingly, the burning objects become more compact, and mutual complementary and perfect flame retardant effect can be brought.

**Figure 6:**
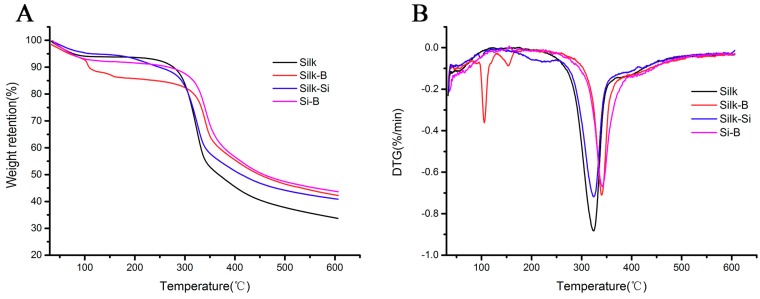
TG (**A**) and DTG (**B**) curves of Silk and Silk-B, Silk-Si and Si-B samples in nitrogen atmosphere.

**Figure 7:**
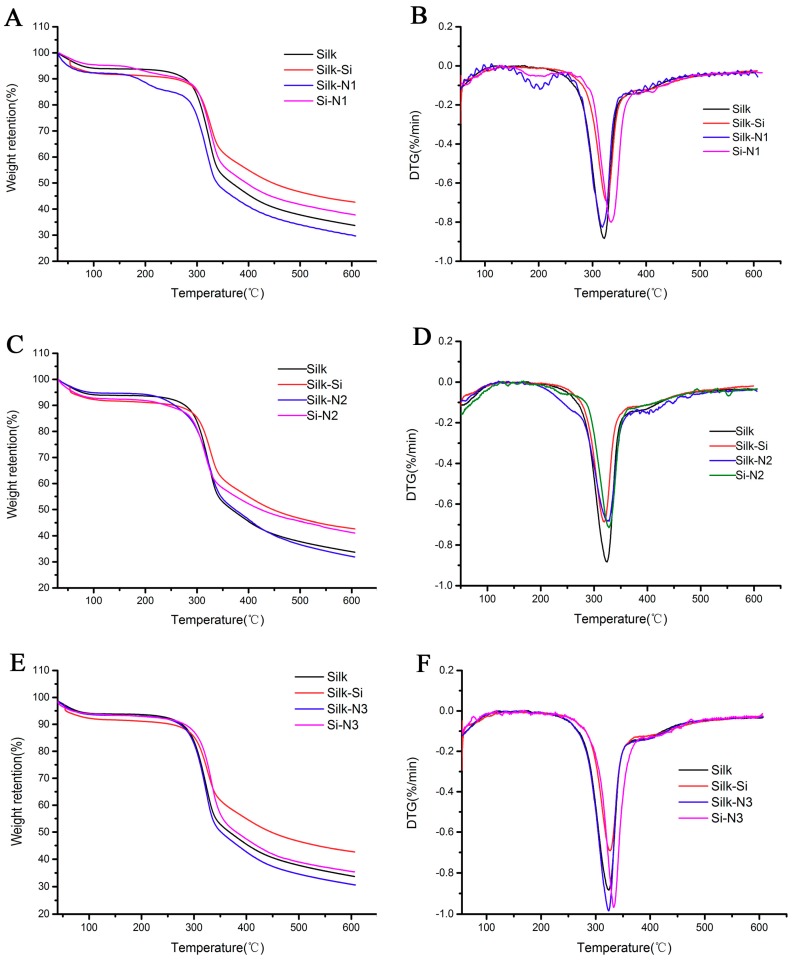
TG (**A**,**C**,**E**) and DTG (**B**,**D**,**F**) curves of Silk and Silk-N_1_, Silk-N_2_ and Silk-N_3_, Si-N_1_ and Si-N_2_, Si-N_3_ samples in nitrogen atmosphere.

**Figure 8:**
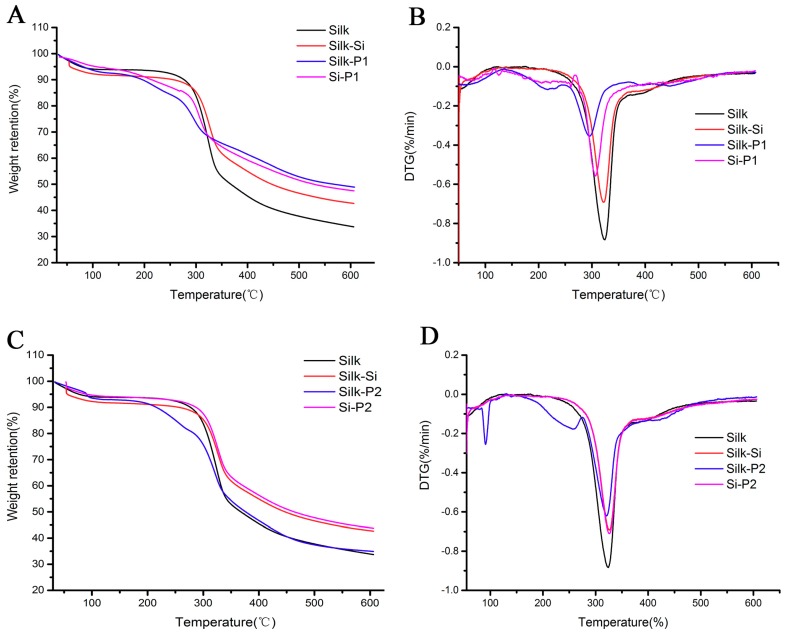
TG (**A**,**C**) and DTG (**B**,**D**) curves of Silk and Silk-P_1_, Silk-P_2_ and Si-P_1_, Si-P_2_ samples in nitrogen atmosphere.

Through the thermogravimetric analysis, the mass change and weight loss rate during the thermal decomposition process can be obtained, and the stability of the combustion process can be evaluated. The TG and DTG (Differential thermal gravity) curves of all samples in nitrogen are shown in Figure 6, Figure 7 and Figure 8.

It can be seen from the figures that the thermal weight loss behavior of the original silk fabric and finished silk fabrics is similar, and the pyrolysis process can be roughly divided into three stages. (i) Six-percent of weight loss before 100 °C was due to the gradual evaporation of adsorbed water on silk fibers. (ii) Through the TG curve, it can be seen that there is no obvious weight loss before 250 °C, and 250–350 °C is the pyrolysis process of silk fabric. During this interval, silk undergoes strong thermal degradation, resulting in the cleavage of silk fibroin macromolecules [30]. The weight loss is significant, which is consistent with the thermal pyrolysis temperature of silk fibers around 300 °C. (iii) After 450 °C, the weight loss rate of the silk gradually stabilized. When the temperature reached 600 °C, the weight loss rate had no obvious change, and the pyrolysis behavior of the silk basically ended. The main occurrence of this stage was the oxidation of carbon. Comparing these curves, it can be seen that the silk fabric treated by the modified silica sol had a lower weight loss rate than the original silk fabric, and the thermal weight loss of the silk fabric finished by the HEDP-doped silica sol was the smallest, indicating that the thermal properties of HEDP-doped silica sol-finished silk fabrics were significantly improved. The order of weight loss of the fabric is Si-P_1_ (HEDP) > Si-P_2_ (DOPO) > Si-B (Boric acid) > Si-N_1_ (Urea) > Si-N_2_ (Cyanoguanidine) > Si-N_3_ (MCA) > original silk.

The fabrics treated with boric acid, urea, HEDP, and DOPO have obvious weight loss in the first stage of combustion (Figure 6, Figure 7A,B and Figure 8). This is because boric acid can release bound water below the combustible temperature and inhibit the formation of combustible gas by changing the thermal decomposition pathway of some combustible materials through cooling and heat absorption [21,31]. Non-combustible gases such as nitrogen and water vapor, and acids (phosphoric acid or polyphosphoric acid) formed during thermal decomposition of HEDP and DOPO, inhibit the thermal cracking reaction in process I to some extent, which further confirms the MCC data. The weight loss of the treated silk fabrics in the first two stages are basically larger than that of the untreated silk fabric, and is smaller than those of the fabrics separately treated by boric acid, urea, HEDP, and DOPO. This is because the treated oxide coating on the surface of silk fibers has a good water retention effect, and the boron, nitrogen and phosphorus flame retardants play a role at this stage, reflecting the matching effect mechanism of silicon-boron, silicon-nitrogen, and silicon complex. In the third stage, since the modified silica sol-finished fabric forms a dense molten coating on the surface of silk fibers, the fabric has a higher weight retention rate and better carbon stability and thermal stability. Table 4 collects the data of relevant parameters in nitrogen atmosphere. In the SE data of TGA (weight residue at 600 °C), SE (Si-N_1_) > SE (Si-N_2_) > SE (Si-P_2_) > 1. It can be concluded that the char formation stability of Si-N_1_ has a synergistic effect, Si-N_2_ and Si-P_2_ sol are only additive (0< SE < 1) effects, while others are only addtive flame retardants.

### 3.4. Pyrolysis-Gas Chromatography/Mass Spectrometry and FT-IR

**Figure 9:**
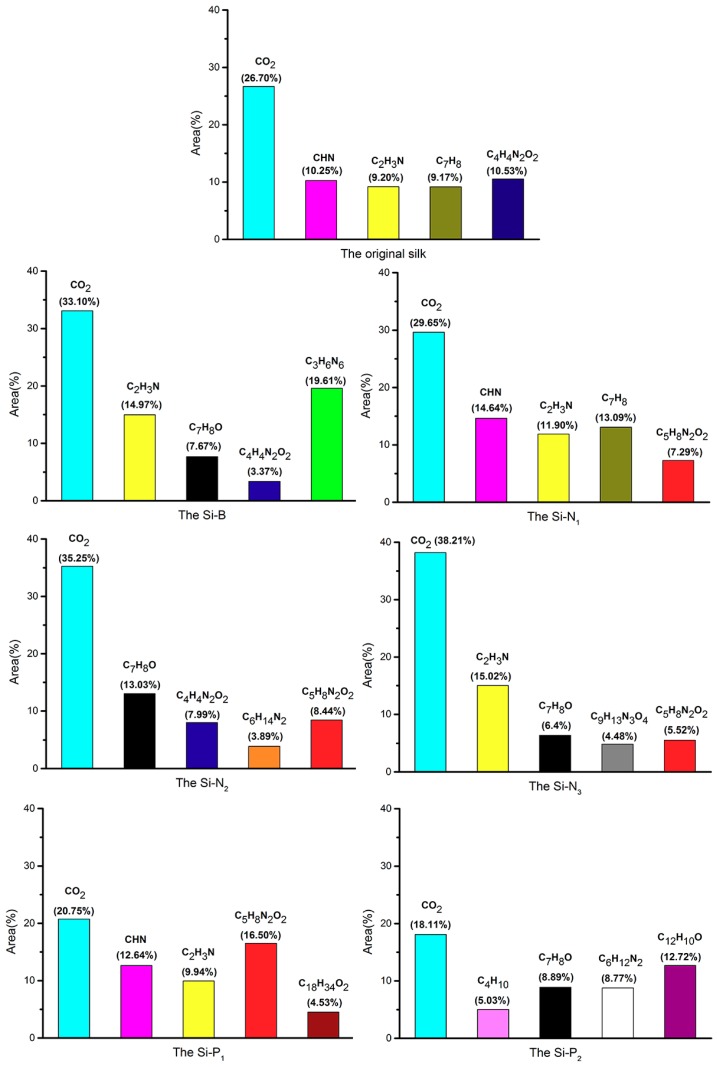
The cleavage productsof original silk and the treated silk fabrics.

Pyrolysis-gas chromatography-mass spectrometry (pyGC-MS) can not only be used to isolate and identify the pyrolysis products, but also to analyze them qualitatively and quantitatively, and to investigate the composition, structure, properties, degradation mechanism, and reaction kinetics [32]. Based on the results of thermogravimetric analysis, pyrolysis temperature was selected at 570 °C and the pyrolysis products and relative contents of the samples at 570 °C were analyzed by pyGC-MS. Figure S1 shows seven pyrolysis-gas chromatography of the original silk (A) and the treated silk (B-G, Si-B, Si-N_1_ and Si-N_2_, Si-N_3_, Si-P_1_, and Si-P_2_, respectively). The collected data about thermal pyrolysis of all samples are summarized in Tables S3–S9. Since the pyrolytic materials are natural polymer materials, the types of cleavage products are diversified and many similar structures appear. Therefore, only the substances with obvious peak types, larger material content, easier to analyze, and representative materials are discussed. Figure 9 shows cleavage products of original silk and the treated silk fabrics.

The thermal pyrolysis order of the chemical bonds on the polymer backbone is directly related to the bond energy [33]. As can be seen from Tables S3–S9, the pyrolysis products produced by original silk and the treated silk are CO_2_ because the bond splitting energy of the C–O bond in the molecular chain is the lowest (86.1 kcal/mol) [34]. In the pyrolysis products, compared with original silk, the types of compounds released from the treated fabric obviously increased. The content of CO_2_ (33.1%) of the Si-B sol increased and the content of flammable lysate decreased (26.01%), and 19.61% of the C_3_H_6_N_6_, a non combustible lysate, was produced. The Si-N sol (Si-N_1_, Si-N_2_, and Si-N_3_) treated fabric also has a higher CO_2_ content (33.1%, 29.65%, 35.25%, respectively), a reduced flammable lysate content, and new lysates such as C_5_H_8_N_2_O_2_, C_6_H_14_N_2_, and C_9_H_13_N_3_O_4_. However, the CO_2_ content of the Si-P sol treated fabric (Si-P_1_, Si-P_2_) was reduced, and new lysates such as C_18_H_34_O_2_, C_4_H_10_, C_6_H_12_N_2_, and C_12_H_10_O were also produced. These results indicate that the pyrolysis method of silk fabrics has changed after treatment. The results show that the splitting way of the fabrics treated by Si-B, Si-N, and Si-P sol is quite different.

**Figure 10:**
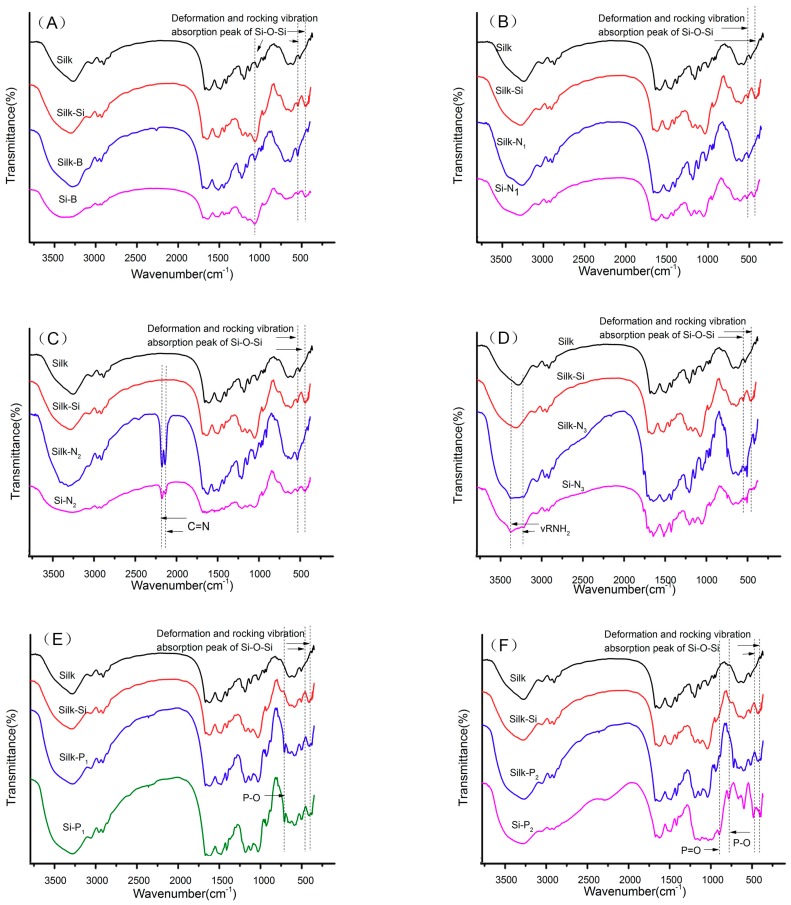
FTIR spectra of all samples. Silk, Silk-Si, Silk-B, Si-B (**A**); Silk, Silk-Si, Silk-N1, Si-N1 (**B**); Silk, Silk-Si, Silk-N2, Si-N2 (**C**); Silk, Silk-Si, Silk-N3, Si-N3 (**D**); Silk, Silk-Si, Silk-P1, Si-P1 (**E**); Silk, Silk-Si, Silk-P2, Si-P2 (**F**).

In the pyrolysis products, compared with the original silk, new compounds such as C_12_H_24_BN, C_12_H_9_O_2_P, and various C-N compounds were produced. Hybrid silica sol was prepared by hydrolytic condensation of tetraethyl orthosilicate with boric acid/urea/cyanoguanidine/MCA/DOPO by the sol-gel method. Combined with infrared spectroscopy (Figure 10) and Table S10 with main peaks description, it can be concluded that boron atoms, nitrogen atoms and phosphorus atoms were introduced through chemical bonding mode. In the silica sol matrix, chemical bonds of Si–O–Si, C=N, Si–NH_2_, B–O, N–H, and P–O were formed. HEDP formed a gel during the hydrolysis condensation reaction with tetraethyl orthosilicate, which was not suitable for finishing silk fabrics. Therefore, HEDP was mixed with the prepared pure silica sol through an ultrasonic oscillator whose action mode with silica sol was physically doping. However, the pyrolysis product of Si was not detected by PyGC-MS, which may be due to the high molecular weight and boiling point of Si compounds, and it is difficult to enter the mass spectrum through the column [35].

### 3.5. Smoke Suppression 

**Figure 11:**
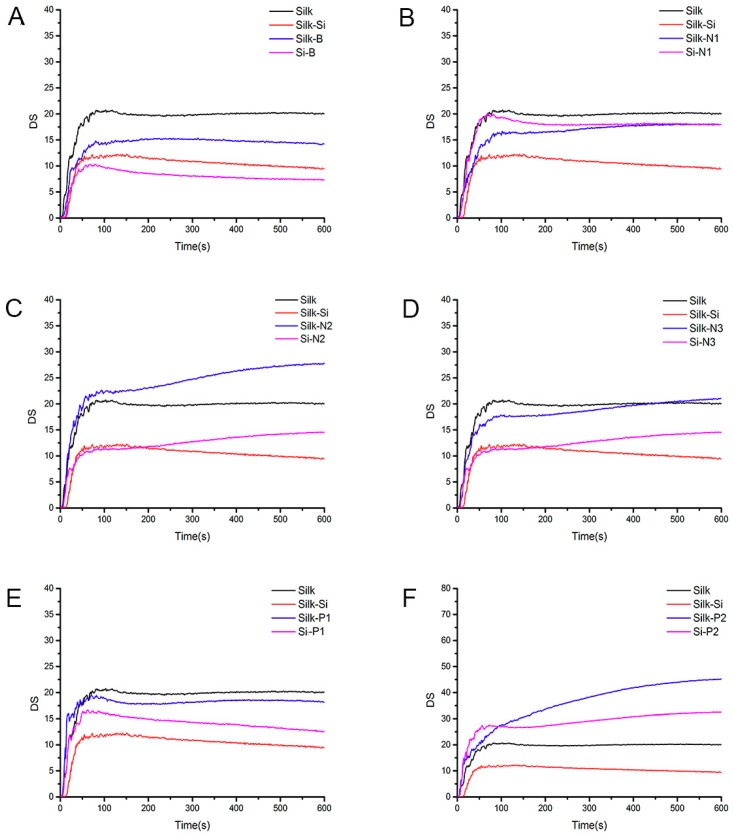
The smoke density curve of all samples. Silk, Silk-Si, Silk-B, Si-B (**A**); Silk, Silk-Si, Silk-N1, Si-N1 (**B**); Silk, Silk-Si, Silk-N2, Si-N2 (**C**); Silk, Silk-Si, Silk-N3, Si-N3 (**D**); Silk, Silk-Si, Silk-P1, Si-P1 (**E**); Silk, Silk-Si, Silk-P2, Si-P2 (**F**).

The smoke suppression performance of materials is of great significance to reduce the poisoning or death caused by heavy smoke in fire. The smoke suppression performance is characterized by the maximum density (D_S_) of the smoke generated during combustion in the smoke density test. The lower the maximum density value generated when the material is burned, the better the smoke suppression performance [36]. The smoke suppression properties of original silk and finished silk fabrics were tested, respectively, and are shown in Figure 11.

Compared with the original fabric (Silk), the smoke density of the finished silk (Silk-B, Silk-B, and Si-B) decreased greatly (Figure 11A). The smoke density of silk fabric, finishing by Si-B sol, was the smallest, and the smoke suppression was the best. This provides good evidence for the respective smoke suppression performance of silicon and boron flame retardants, and also reflects the synergistic effect of silicon and boron on smoke suppression performance. However, the smoke density off Silk-N_2_ and Silk-P_2_ samples increased a lot. While the smoke density of samples Silk-N_1_, Silk-N_3_, and Silk-P_1_ was lower than that of the original silk, it was much higher than that of pure silica-sol (Silk-Si). This is because nitrogen flame retardants (especially cyanoguanidine) can decompose ammonia, nitrogen, nitrogen oxides, water vapor, and other noncombustible gases after heating [37]. The organophosphorus flame retardants (especially DOPO) produced phosphoric acid, polyphosphoric acid, and water vapor during the combustion process, which diluted the oxygen required for the combustion process and takes away most of the heat in the process. However, the generated gas was mixed with air and condensed to form spherical suspended soot, which increased the density of the smoke. The density of smoke of silica-nitrogen sol and silicon-phosphorus sol treated silk fabric (Si-N_1_, Si-N_2_ and Si-N_3_, Si-P_1_ and Si-P_2_) decreased a lot. While it was higher than pure silica sol (Silk-Si), it was much lower than the original silk fabric. This is a good evidence for the synergistic effect of silicon-nitrogen and silicon-phosphorus hybrid sols in suppressing smoke. The reason is mainly that silicon has good smoke suppressing property. At high temperatures, the sol forms an oxide-insulating layer of silicon and nitrogen, which effectively prevents the release of smoke and greatly reduces the generation of smoke.

## 4. Conclusions

In this work, the modified silica sol was applied in silk fabric finish to obtain flame retardantcy. Through the relevant performance tests on the finished silk samples, the flame retardant effects of different modified silica sol systems on silk fabrics were analyzed and the synergistic effects in the system were discussed. The main conclusions are as follows:

(1) It can be confirmed from SEM and EDS tests that the flame retardant was finished onto the silk fiber, and the silica sol system can form a dense film on the surface of the silk fabric.
(2) LOI results indicated the synergistic effect (SE > 1) in the flame retardant system of Si-B, Si-N, and Si-N_3_, Si-P_2_, and the synergistic effect of Si-N_3_ was the most obvious. The Si-N_2_ and Si-P_1_ flame retardant systems only showed cumulative effects (0 < SE < 1). The data of the damaged length in vertical combustion proved that Si-B, Si-N_1_, and Si-N_3_ (combined with LOI results) still had a synergistic effect, and Si-P_1_ also showed a weak synergy, indicating that the results obtained from LOI and vertical combustion were basically similar. Si-B not only had a synergistic effect on improving the flame retardancy of silk fabric, but also showed synergy in reducing the heat release rate of fabric combustion. In the results of TGA, there was a synergistic effect between Si-N_1_, Si-N_2_, and Si-P_2_ on the char formation stability, and the others were only additive effects. Silicon and boron flame retardants individually had certain smoke suppression performance. The silicon-boron sol had a good synergistic effect on smoke suppression. The silicon-nitrogen and silicon-phosphorus sols had a certain synergistic effect on smoke suppression. Generally, in terms of flame retardancy (Fr), Si-P > Si-B > Si-N. Since the Fr of flame retardant containing P was very good, the Fr of the flame retardant containing Si, B, and N was relatively poor, and Si-B and Si-N were better than Si-P in synergistic effect.
(3) Combined with the results of all tests, the flame retardancy mechanism of the sol system was summarized as follows. Silicon-boron (Si-B) sol: At the beginning of combustion, the bound water released by boric acid acts to cool and absorb heat. The silicon molecules are cleaved into carbon at high temperature, thereby increasing the oxidation resistance of the carbon layer. The Si-B silica sol melts and forms a molten coating to prevent combustion. It plays a major role in the II and III processes during the silk combustion cycle. Silicon-nitrogen (Si-N) sol: The Si-N sol has endothermic reaction such as phase change and dehydration at high temperature, and melts into molten covering layer. The non-combustible gases decomposed by nitrogen containing flame retardants also plays a role of flame retardancy. The Si-N sol plays a major role in the I, II, and III processes in the silk combustion cycle. Silicon-phosphorus (Si-P) sol: At high temperature, phosphorus can form acid to promote the formation of carbon layer, while silicon will increase the thermal stability of the carbon layer. There is also a molten cover layer formed by the degradation of the Si-P sol. It plays a major role in the I, II, and III processes in the silk combustion cycle.

## Figures and Tables

**Table 1:** Elemental analysis of the pure silk and the treated silk fabrics.

Samples	Element Content (wt.%)					
	C [%]	N [%]	O [%]	Si [%]	B [%]	P [%]
Silk	63.62 ± 3.68	14.15 ± 1.65	22.23 ± 2.13	-	-	-
Si-B	60.86 ± 2.45	11.02 ± 1.35	19.29 ± 2.42	1.04 ± 0.46	7.79 ± 1.25	-
Si-N_1_	57.09 ± 3.39	14.07 ± 0.95	26.67 ± 1.95	2.17 ± 0.15	-	-
Si-N_2_	60.87 ± 4.67	14.00 ± 1.54	23.78 ± 1.78	1.35 ± 0.27	-	-
Si-N_3_	58.03 ± 2.59	17.37 ± 1.36	23.19 ± 2.34	1.41 ± 0.31	-	-
Si-P_1_	57.85 ± 3.13	10.17 ± 1.47	27.04 ± 3.46	1.21 ± 0.22	-	3.73 ± 0.21
Si-P_2_	66.70 ± 4.10	8.84 ± 0.85	22.11 ± 2.15	1.83 ± 0.20	-	0.52 ± 0.07

**Table 2:** LOI and the vertical burning results for all samples.

Sample	LOI (%)	Damaged Length (cm)
Silk	24.00 ± 0.31	30.00 ± 0.51
Silk-Si	26.10 ± 0.26	25.90 ± 0.32
Silk-B	28.00 ± 0.15	15.80 ± 0.23
Silk-N_1_	25.50 ± 0.33	24.60 ± 0.20
Silk-N_2_	25.30 ± 0.22	19.00 ± 0.38
Silk-N_3_	25.40 ± 0.35	21.00 ± 0.22
Silk-P_1_	40.10 ± 0.39	14.90 ± 0.19
Silk-P_2_	31.00 ± 0.20	15.50 ± 0.26
Si-B	31.20 ± 0.18	9.80 ± 0.11
Si-N_1_	29.70 ± 0.26	19.50 ± 0.34
Si-N_2_	27.00 ± 0.31	19.20 ± 0.48
Si-N_3_	29.80 ± 0.28	17.00 ± 0.50
Si-P_1_	38.60 ± 0.22	9.90 ± 0.19
Si-P_2_	33.50 ± 0.32	14.80 ± 0.24
SE ^a^ (Si-B)	1.18 ± 0.06	1.10 ± 0.14
SE ^a^ (Si-N_1_)	1.58 ± 0.12	1.11 ± 0.15
SE ^a^ (Si-N_2_)	0.88 ± 0.18	0.72 ± 0.13
SE ^a^ (Si-N_3_)	1.65 ± 0.09	1.00 ± 0.09
SE ^a^ (Si-P_1_)	0.80 ± 0.13	1.05 ± 0.14
SE ^a^ (Si-P_2_)	1.04 ± 0.15	0.82 ± 0.07

^a^ Synergistic effectiveness.

**Table 3:** Characteristic parameter of the micro calorimeter test for all samples.

Sample	HRC (J/g·k)	pHRR (W/g)	THR (kJ/g)	Tmax (°C)
Silk	147.00 ± 1.10	141.30 ± 2.31	8.80 ± 0.29	320.30 ± 0.59
Silk-Si	113.00 ± 0.91	104.00 ± 1.98	8.10 ± 0.51	297.50 ± 0.67
Silk-B	144.00 ± 0.82	132.50 ± 1.15	14.40 ± 0.64	351.00 ± 0.58
Silk-N_1_	148.00 ± 0.56	130.30 ± 3.24	17.10 ± 0.39	327.60 ± 0.38
Silk-N_2_	140.00 ± 0.49	127.20 ± 2.58	15.40 ± 0.37	333.10 ± 0.45
Silk-N_3_	139.00 ± 0.79	123.50 ± 2.64	10.40 ± 0.24	305.20 ± 0.61
Silk-P_1_	60.00 ± 1.39	49.00 ± 1.59	10.30 ± 0.56	303.10 ± 0.57
Silk-P_2_	115.00 ± 0.86	105.30 ± 1.87	18.70 ± 0.49	332.70 ± 0.43
Si-B	91.00 ± 0.79	88.70 ± 1.23	6.20 ± 0.48	308.50 ± 0.54
Si-N_1_	124.00 ± 0.45	113.70 ± 1.84	13.70 ± 0.73	340.60 ± 0.66
Si-N_2_	123.00 ± 0.61	110.40 ± 1.45	10.20 ± 0.68	290.00 ± 0.71
Si-N_3_	108.00 ± 0.39	100.50 ± 1.97	9.40 ± 0.18	334.20 ± 0.84
Si-P_1_	57.00 ± 0.94	45.33 ± 2.58	9.80 ± 0.26	295.50 ± 0.63
Si-P_2_	90.00 ± 0.67	84.40 ± 2.46	11.10 ± 0.57	334.40 ± 0.75
SE ^a^ (Si-B)	1.51 ± 0.11	1.41 ± 0.12	0.53 ± 0.12	-
SE ^a^ (Si-N_1_)	0.70 ± 0.06	0.56 ± 0.13	0.64 ± 0.09	-
SE ^a^ (Si-N_2_)	0.24 ± 0.25	0.29 ± 0.19	0.86 ± 0.20	-
SE ^a^ (Si-N_3_)	0.22 ± 0.21	0.55 ± 0.23	0.72 ± 0.18	-
SE ^a^ (Si-P_1_)	0.74 ± 0.18	0.74 ± 0.20	1.25 ± 0.21	-
SE ^a^ (Si-P_2_)	0.79 ± 0.23	0.73 ± 0.18	0.27 ± 0.14	-

^a^ Synergistic effectiveness.

**Table 4:** TGA data in nitrogen atmosphere.

Sample	T_10%_ (°C)	T_50%_ (°C)	Weight Residue at 600 °C (%)
Silk	277.04	367.71	33.95
Silk-Si	248.91	412.94	41.08
Silk-B	114.33	462.06	43.55
Silk-N_2_	257.07	373.98	32.14
Silk-P_2_	215.84	377.10	35.18
Si-B	272.47	459.54	44.02
Si-N_1_	245.30	422.93	42.17
Si-N_2_	240.95	423.78	41.39
Si-N_3_	282.23	380.94	35.69
Si-P_1_	214.07	529.19	47.76
Si-P_2_	287.22	463.74	44.04
SE ^a^ (Si-B)	-	-	0.60
SE ^a^ (Si-N_1_)	-	-	2.56
SE ^a^ (Si-N_2_)	-	-	1.40
SE ^a^ (Si-N_3_)	-	-	0.43
SE ^a^ (Si-P_1_)	-	-	0.62
SE ^a^ (Si-P_2_)	-	-	1.22

^a^ Synergistic effectiveness.

## Supplementary Materials

The following are available online at http://www.mdpi.com/1996-1944/11/10/1842/s1, Figure S1: The Pyrolysis gas chromatography of the original silk and finished silk (Si-B, Si-N_1_ and Si-N_2_, Si-N_3_, Si-P_1_ and Si-P_2_), Table S1: Name, code and chemical structures of sol-gel precursor and synergist agents, Table S2: Total dry solids add-on of sols on silk samples, Table S3: The pyrolysis gas chromatography of Silk, Table S4: The pyrolysis gas chromatography of Si-B, Table S5: The pyrolysis gas chromatography of Si-N_1_, Table S6: The pyrolysis gas chromatography of Si-N_2_, Table S7: The pyrolysis gas chromatography of Si-N_3_, Table S8: The pyrolysis gas chromatography of Si-P_1_, Table S9: The pyrolysis gas chromatography of Si-P_2_, Table S10: The main peaks description about FTIR spectra of all samples.
