# Supplementary material for: Synergistic Effects and Mechanism of Modified Silica Sol Flame Retardant Systems on Silk Fabric
Source: Materials (Basel). 2018 Sep 27;11(10):1842. doi: 10.3390/ma11101842 (PMC6212991; doi:10.3390/ma11101842)
Supplement: Supplementary file 1 [file materials-11-01842-s001.pdf]

Synergistic effects and mechanism of modified silica sol flame retardant systems on silk fabric

Chun Liu, Tieling Xing*, Bingju Wei, Guoqiang Chen

National Engineering Laboratory for Modern Silk, College of Textile and Clothing Engineering, Soochow University, Suzhou 215123, China

* Correspondence: xingtieling@suda.edu.cn; Tel.: 86-512-67061152

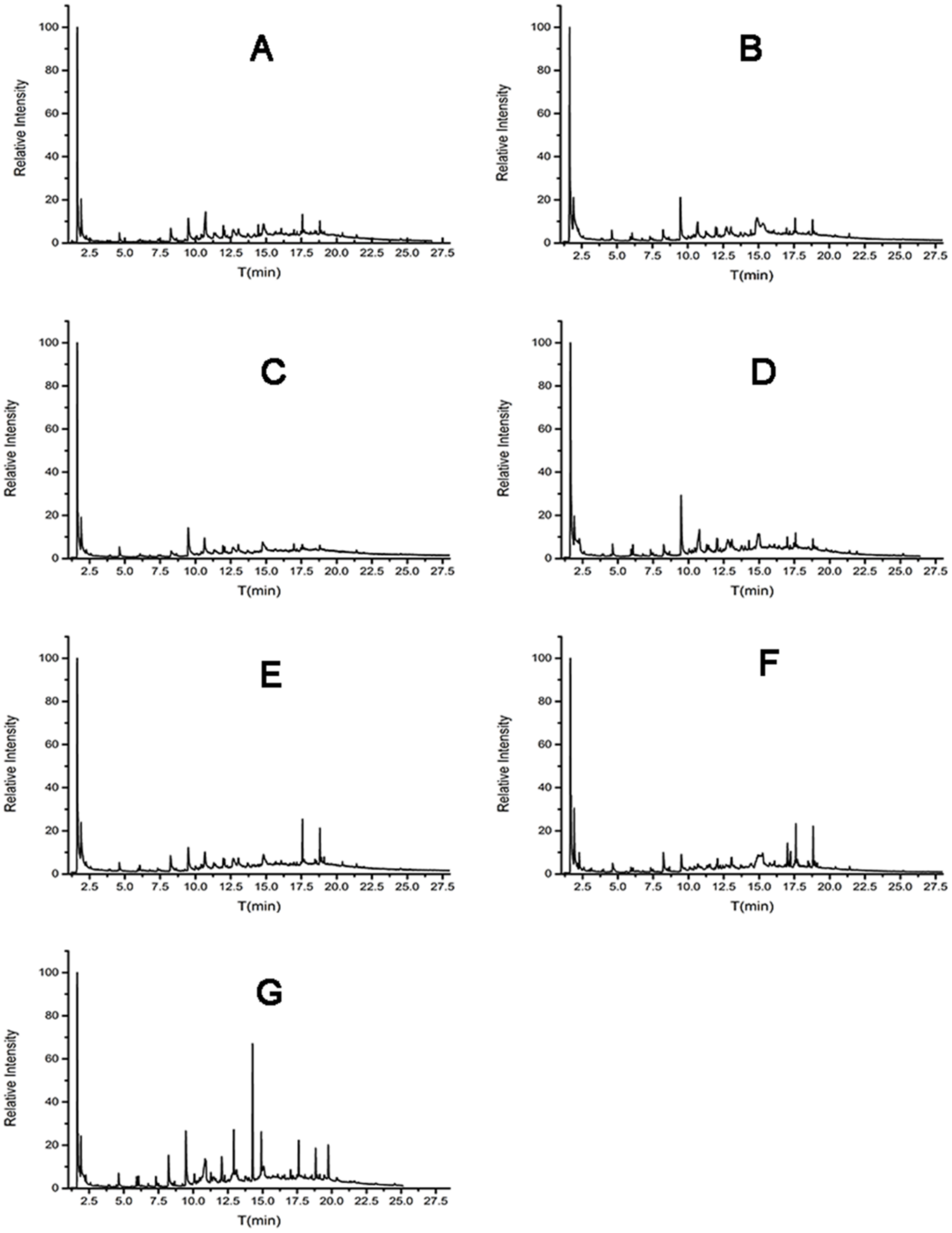

Figure S1. The Pyrolysis gas chromatography of the original silk and finished silk (Si-B, Si-N_1 and Si-N_2 ,Si-N_3, Si-P_1 and Si-P_2).

Table S1. Name, code and chemical structures of sol-gel precursor and synergist agents.

Name	Code	Chemical Structure
Tetraethyl orthosilicate	Si	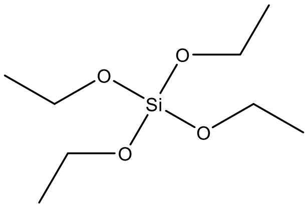
Boric acid	B.	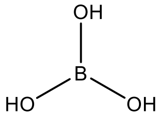
Urea	N_1	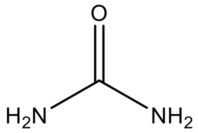
Dicyandiamide	N_2	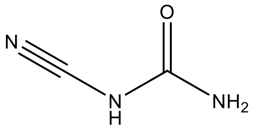
Melamine cyanurate	N_3	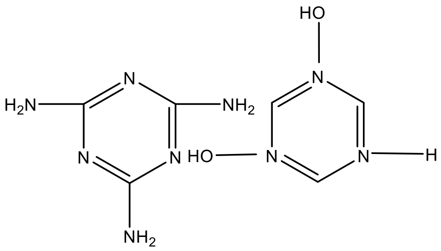
1-hydroxyethane 1,1-diphosphonic acid	P_1	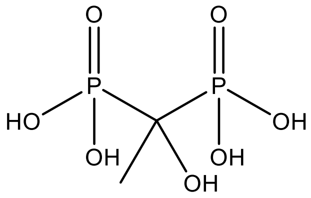

6H-dibenz(C,E)(1,2)oxaphosphorin-6-oxide	P_2	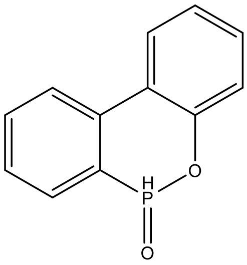

Table S2. Total dry solids add-on of sols on silk samples.

Sample	Add-on (wt.-%)	Sample	Add-on (wt.-%)
Silk	-	Silk-P_2	33.04 ± 0.22
Silk-Si	27.44 ± 0.24	Si-B	19.72 ± 0.18
Silk-B	13.03 ± 0.16	Si-N_1	10.98 ± 0.11
Silk-N_1	2.71 ± 0.09	Si-N_2	22.09 ± 0.20
Silk-N_2	7.70 ± 0.23	Si-N_3	28.11 ± 0.15
Silk-N_3	4.86 ± 0.12	Si-P_1	26.62 ± 0.23
Silk-P_1	23.01 ± 0.26	Si-P_2	33.71 ± 0.19

Table S3. The pyrolysis gas chromatography of Silk.

No.	Time (min)	Compound Name	Area (%)	No.	Time (min)	Compound Name	Area (%)
1	1.634	CO_2	26.70	12	10.715	$C_4H_4N_2O_2$	10.53
2	1.686	CHN	10.25	13	11.972	$C_7H_{13}NO_2$	2.69
3	1.9	C_2H_3N	9.20	14	12.065	C_8H_7N	1.42
4	2.263	C_3H_5N	0.56	15	12.668	$C_9H_{13}N_3O_4$	5.35
5	2.589	C_4H_7N	0.27	16	13.043	$C_8H_{10}O_2$	2.26
6	3.868	C_5H_9N	0.17	17	14.446	$C_{12}H_{24}O_2$	1.31
7	3.976	C_5H_9N	0.20	18	14.818	$C_5H_8N_2O_2$	5.53
8	4.614	C_7H_8	2.02	19	16.074	$C_{14}H_{28}O_2$	1.10
9	6.07	$C_7H_{13}N$	0.43	20	16.978	$C_{11}H_{12}FNO$	0.81
10	8.248	C_6H_6O	5.18	21	17.575	$C_{16}H_{32}O_2$	2.06
11	9.489	C_7H_8O	9.17	22	18.81	$C_{18}H_{34}O_2$	2.79

Table S4. The pyrolysis gas chromatography of Si-B.

No.	Time (min)	Compound Name	Area (%)	No.	Time (min)	Compound Name	Area (%)
1	1.645	CO_2	33.31	16	10.415	$C_6H_{11}ClO_2$	0.52
2	1.913	C_2H_3N	14.97	17	10.692	$C_4H_4N_2O_2$	3.37
3	2.27	C_3H_5N	0.39	18	11.266	C_8H_8O	0.80
4	2.603	C_4H_7N	0.24	19	11.968	$C_7H_{13}NO_2$	1.15
5	3.88	C_5H_9N	0.15	20	12.054	C_8H_7N	1.06
6	3.982	C_5H_9N	0.13	21	12.728	$C_9H_{13}N_3O_4$	3.03
7	4.625	C_7H_8	1.62	22	13.046	$C_7H_{10}N_2O$	1.54
8	5.93	C_5H_7N	0.46	23	13.729	$C_6H_{10}N_2O_2$	0.81
9	6.062	$C_7H_{13}N$	0.78	24	14.446	$C_{12}H_{24}O_2$	0.39
10	6.763	C_8H_8	0.23	25	14.892	$C_3H_6N_6$	19.61
11	7.32	C_6H_9N	0.35	26	16.983	$C_{12}H_{24}BN$	0.87
12	8.248	C_6H_6O	2.49	27	17.582	$C_{16}H_{32}O_2$	1.52
13	8.531	$C_9H_{14}N_2$	0.09	28	18.816	$C_{18}H_{34}O_2$	1.85
14	8.659	$C_7H_{11}N$	0.19	29	21.408	$C_{18}H_{33}N$	0.41
15	9.469	C_7H_8O	7.67				

Table S5. The pyrolysis gas chromatography of Si-N1.

No.	Time (min)	Compound Name	Area (%)	No.	Time (min)	Compound Name	Area (%)
1	1.628	CO_2	29.65	11	10.637	$C_4H_4N_2O_2$	4.93
2	1.682	CHN	14.64	12	11.937	$C_6H_{11}NO_4$	1.45
3	1.9	C_2H_3N	11.90	13	12.064	C_8H_7N	1.39
4	2.13	C_4H_8O	0.37	14	12.661	$C_9H_{13}N_3O_4$	3.65
5	2.262	C_3H_5N	0.73	15	13.026	$C_8H_{10}O_2$	1.79
6	2.593	C_4H_7N	0.27	16	14.753	$C_5H_8N_2O_2$	7.29
7	4.614	C_7H_8	2.40	17	16.967	$C_8H_{11}N_5O$	1.33
8	6.017	$C_7H_{13}N$	0.61	18	17.566	$C_{16}H_{32}O_2$	0.56
9	8.289	C_6H_6O	2.86	19	18.804	$C_{18}H_{34}O_2$	1.09
10	9.478	C_7H_8O	13.09				

Table S6. The pyrolysis gas chromatography of Si-N_2.

No.	Time (min)	Compound Name	Area (%)	No.	Time (min)	Compound Name	Area (%)
1	1.641	CO_2	35.28	19	10.434	$C_5H_7NO_2$	1.00
2	1.908	C_2H_3N	3.67	20	10.782	$C_4H_4N_2O_2$	7.99
3	2.265	C_3H_5N	1.20	21	11.287	C_8H_8O	1.42
4	2.597	C_4H_7N	0.35	22	11.424	$C_8H_{20}BN$	0.60
5	3.877	C_5H_9N	0.16	23	12.008	$C_{11}H_{22}N_2$	1.77
6	3.979	C_5H_9N	0.21	24	12.05	C_8H_7N	1.37
7	4.47	C_4H_5N	0.27	25	12.772	$C_6H_{14}N_2$	3.89
8	4.622	C_7H_8	1.87	26	12.922	$C_{12}H_{10}$	0.47
9	5.924	C_5H_7N	0.82	27	13.003	C_9H_9N	0.51
10	6.058	$C_7H_{13}N$	1.09	28	13.073	$C_7H_{10}N_2O$	1.29
11	6.765	C_8H_8	0.21	29	13.745	$C_8H_{17}NO$	1.52
12	7.315	C_6H_9N	0.99	30	13.998	C_8H_7NO	0.83
13	7.503	C_6H_9N	0.16	31	14.287	$C_{12}H_{10}O$	1.03
14	8.243	C_6H_6O	2.23	32	14.994	$C_5H_8N_2O_2$	8.44
15	8.527	$C_9H_{14}N_2$	0.15	33	17.003	$C_8H_{11}N_5O$	1.39
16	8.657	$C_7H_{11}N$	0.26	34	17.464	$C_9H_{14}O_2$	0.94
17	9.468	C_7H_8O	13.03	35	17.586	$C_{16}H_{32}O_2$	1.56
18	10.041	$C_6H_8O_2$	0.74	36	18.816	$C_{18}H_{34}O_2$	1.29

Table S7. The pyrolysis gas chromatography of Si-N_3.

No.	Time (min)	Compound Name	Area (%)	No.	Time (min)	Compound Name	Area (%)
1	1.621	CO_2	38.21	14	11.295	C_8H_8O	0.75
2	1.889	C_2H_3N	15.02	15	11.97	$C_7H_{13}NO_2$	1.55
3	2.127	C_4H_8O	0.97	16	12.055	C_8H_7N	1.11
4	2.248	C_3H_5N	0.92	17	12.674	$C_9H_{13}N_3O_4$	4.84
5	4.61	C_7H_8	1.38	18	13.036	$C_8H_{10}O_2$	1.82
6	5.924	C_5H_7N	0.47	19	14.807	$C_5H_8N_2O_2$	5.52
7	6.054	$C_6H_8O_2$	0.74	20	17.581	$C_{16}H_{32}O_2$	4.56
8	7.319	C_6H_9N	0.80	21	18.466	$C_{19}H_{38}O_2$	0.37
9	8.233	C_6H_6O	3.33	22	18.815	$C_{18}H_{34}O_2$	4.65
10	9.481	C_7H_8O	6.40	23	18.934	$C_{18}H_{36}O_2$	0.46
11	10.006	$C_4H_8N_4$	0.74	24	19.116	$C_{16}H_{33}NO$	0.58
12	10.407	$C_7H_{10}N_2$	0.46	25	20.403	$C_{18}H_{35}NO$	0.45
13	10.672	$C_4H_4N_2O_2$	3.90				

Table S8. The pyrolysis gas chromatography of Si-P_1.

No.	Time (min)	Compound Name	Area (%)	No.	Time (min)	Compound Name	Area (%)
1	1.635	CO_2	20.75	26	11.525	$C_6H_{11}NO$	0.75
2	1.687	CHN	12.64	27	11.979	$C_{11}H_{22}N_2$	0.63
3	1.902	C_2H_3N	9.94	28	12.049	C_8H_7N	1.22
4	2.138	C_4H_8O	0.56	29	12.18	$C_9H_{16}O$	0.25
5	2.259	C_3H_5N	1.55	30	12.25	$C_{10}H_{16}O$	0.34
6	2.538	$C_2H_4O_2$	0.21	31	12.437	$C_6H_9N_3O$	0.43
7	2.593	C_4H_7N	0.20	32	13.06	$C_8H_{10}O_2$	2.02
8	3.006	$C_5H_{10}O$	0.14	33	15.241	$C_5H_8N_2O_2$	16.50
9	3.127	C_4H_7N	0.33	34	15.74	$C_{16}H_{29}NO_4$	0.91
10	3.873	C_5H_9N	0.16	35	16.094	$C_{14}H_{28}O_2$	0.32
11	3.974	C_5H_9N	0.22	36	17.015	$C_8H_{11}N_5O$	3.14
12	4.629	C_7H_8	2.33	37	17.17	$C_{16}H_{31}N$	0.45
13	5.563	$C_7H_{11}NO$	0.11	38	17.225	$C_8H_{13}N_7$	1.50
14	5.922	C_5H_7N	0.50	39	17.53	$C_8H_{13}N_7$	0.38
15	6.066	$C_7H_{13}N$	0.44	40	17.604	$C_{16}H_{32}O_2$	4.11
16	7.316	C_6H_9N	0.68	41	17.711	$C_{14}H_{23}NO$	0.70
17	7.501	C_6H_9N	0.16	42	17.787	$C_{18}H_{36}O_2$	0.15
18	8.221	C_6H_6O	3.23	43	18.47	$C_{18}H_{33}N$	0.41

19	8.526	$C_7H_{11}N$	0.22	44	18.584	$C_{17}H_{37}N$	0.27
20	8.655	$C_7H_{11}N$	0.28	45	18.829	$C_{18}H_{34}O_2$	4.53
21	9.482	C_7H_8O	2.84	46	18.945	$C_{18}H_{36}O_2$	0.52
22	10.42	$C_5H_7NO_2$	0.51	47	18.987	$C_{20}H_{38}O_2$	0.31
23	10.648	$C_4H_7N_3O$	094	48	19.123	$C_{16}H_{33}NO$	0.26
24	11.293	C_8H_8O	0.44	49	21.407	$C_{18}H_{33}N$	0.29
25	11.423	C_9H_9N	0.23				

Table S9. The pyrolysis gas chromatography of Si-P_2.

No.	Time (min)	Compound Name	Area (%)	No.	Time (min)	Compound Name	Area (%)

1	1.636	CO_2	18.11	20	8.527	$C_9H_{14}N_2$	0.17
2	1.703	C_4H_{10}	5.03	21	8.656	$C_7H_{11}N$	0.20
3	1.801	C_2H_6O	0.97	22	9.227	C_7H_8O	0.24
4	1.899	C_2H_3N	3.63	23	9.468	C_7H_8O	8.89
5	2.257	C_3H_5N	0.58	24	10.089	$C_7H_{12}O_2$	0.75
6	2.59	C_4H_7N	0.28	25	10.862	$C_6H_{12}N_2$	8.77
7	3.127	C_4H_7N	0.11	26	11.28	C_8H_8O	1.54
8	3.871	C_5H_9N	0.15	27	12.056	C_8H_7N	3.62
9	3.975	C_5H_9N	0.15	28	12.266	$C_{13}H_{10}F_6N_2O_7$	0.72
10	4.461	C_4H_5N	0.23	29	12.926	$C_{12}H_{10}$	3.81
11	4.62	C_7H_8	1.71	30	13.112	$C_8H_{10}O_2$	2.84
12	5.919	C_5H_7N	0.76	31	14.289	$C_{12}H_{10}O$	12.72
13	6.057	$C_7H_{13}N$	0.76	32	14.909	$C_{13}H_{10}$	3.49
14	6.271	C_8H_{10}	0.09	33	15.074	$C_5H_8N_2O_2$	2.95
15	6.762	C_8H_8	0.22	34	17.028	$C_8H_{11}N_5O$	0.94
16	7.311	C_6H_9N	0.64	35	17.616	$C_{16}H_{32}O_2$	3.03
17	7.362	C_6H_9N	0.21	36	18.84	$C_{18}H_{34}O_2$	3.03
18	7.501	C_6H_9N	0.23	37	19.464	$C_{12}H_9O_2P$	1.09
19	8.218	C_6H_6O	3.83	38	19.75	$C_{12}H_9NO_3$	3.51

Table S10. The main peaks description about FTIR spectra of all samples.

Figure 9	Main Peaks Description
All Samples	The telescopic vibration absorption peak near 2930 cm^{-1} was -OH contained in silk fabric.<br>The Vibration absorption peak near 1640 cm^{-1}, 1500 cm^{-1} and 1235 cm^{-1} were amide I, the amide II and amide III in the silk fabric.<br>The 3500 cm^{-1} ~ 3000 cm^{-1} was a strong -NH- absorption peak in the silk fabric structure.
A	The deformation and rocking vibration absorption peaks of Si-O-Si appeared at 430 cm^{-1} and 547 cm^{-1}, respectively.<br>The asymmetric stretching vibration absorption peak of Si-O appeared at 1077cm^{-1}.<br>This indicates that the silica sol has formed a Si-O-Si bonding structure on the surface of the fabric.
B	The deformation and rocking vibration absorption peaks of Si-O-Si appeared at 436 cm^{-1} and 521 cm^{-1}, respectively.<br>The telescopic vibration absorption peak of B-O appeared at 1443 cm^{-1}

C	The deformation and rocking vibration absorption peaks of Si-O-Si appeared at 432 cm^{-1} and 547 cm^{-1}, respectively. The vibration absorption peak of C=N appeared at 2194 cm^{-1} and 2149 cm^{-1}, respectively.
D	The deformation and rocking vibration absorption peaks of Si-O-Si appeared at 475 cm^{-1} and 573 cm^{-1}, respectively. The telescopic vibration absorption peak of Si-NH_2 appeared at 3241 cm^{-1} and 3384 cm^{-1}, respectively.
E	The deformation and rocking vibration absorption peaks of Si-O-Si appeared at 449 cm^{-1} and 508 cm^{-1}, respectively. The vibration absorption peak of P-O appeared at 734 cm^{-1}.
F	The deformation and rocking vibration absorption peaks of Si-O-Si appeared at 462 cm^{-1} and 509 cm^{-1}, respectively. The vibration absorption peak of P-O appeared at 762 cm^{-1} and 852 cm^{-1}, respectively
